# The Metric System

**Published:** 1896-04

**Authors:** 


In the opinion of *Engineering*, London, the compulsory adoption of the metric system of weights and measures two years after the passage of the law to that effect, as recently recommended by a parliamentary committee, will be enormously beneficial. It says: "The time has gone past when it is necessary to furnish arguments as to the advantages of the metric system over present confused methods. Those whose business it is to deal with foreign countries know best how much they lose when they come into competition with manufacturers from Germany and Belgium, from the inability or indisposition of other nations to comprehend British standards." The paper, which is a recognized authority, considers it certain that the metric system will be adopted in England.—*Literary Digest.*

The oldest medical recipe is said, by a French medical journal, to be that of a hair-tonic for an Egyptian queen. It is dated 4,000 B. C., and directs that dogs' paws and asses' hoofs be boiled with dates in oil.—*Literary Digest.*

The latest antiseptic, produced in Germany, is *potassiumorthodinitrocresolate.* It is odorless and cheap, and is said to be destructive to all bacteria. A portion of the potency of this substance is supposed to reside in the name.—*Atlanta Med. and Surg. Journal.*

